# Yellow Fever Virus (YFV) Detection in Different Species of Culicids Collected During an Outbreak in Southeastern Brazil, 2016–2019

**DOI:** 10.3390/tropicalmed10050118

**Published:** 2025-04-24

**Authors:** Giovana Santos Caleiro, Lucila Oliveira Vilela, Karolina Morales Barrio Nuevo, Rosa Maria Tubaki, Regiane Maria Tironi de Menezes, Luis Filipe Mucci, Juliana Telles-de-Deus, Eduardo Sterlino Bergo, Emerson Luiz Lima Araújo, Mariana Sequetin Cunha

**Affiliations:** 1 Center of Virology, Adolfo Lutz Institute, São Paulo 01246-902, Brazil; giovanacaleiro@gmail.com (G.S.C.); karolinamorales182@gmail.com (K.M.B.N.); 2 Cuny Graduate School of Public Health, New York, NY 10065, USA; lucilavilela@icloud.com; 3 Pasteur Institute, São Paulo 05508-020, Brazil; tubaki.rm@gmail.com (R.M.T.); rmtironi@gmail.com (R.M.T.d.M.); julianatd77@gmail.com (J.T.-d.-D.); 4 Pasteur Institute, Taubaté 12010-200, Brazil; lfmucci@gmail.com; 5 Pasteur Institute, Ribeirão Preto 14020-900, Brazil; edusteber@uol.com.br; 6 Brazilian Ministry of Health, Brasília 70719-040, Brazil; emerson.araujo@saude.gov.br

**Keywords:** yellow fever virus, surveillance, Brazil, RT-qPCR, vector transmission

## Abstract

Yellow fever virus (YFV) is an endemic arbovirus in parts of Africa and the Americas. In Brazil, following the eradication of the urban transmission cycle, YFV is maintained in a sylvatic cycle involving several species of neotropical primates and mosquitoes of the genera Haemagogus and Sabethes, which serve as primary and secondary vectors, respectively. During the 2016–2019 outbreak in São Paulo State, a total of 3731 mosquito pools were collected from sites with ongoing epizootic events in 192 municipalities. The RT-qPCR analysis detected YFV in 46 pools (1.4%) across nine mosquito species, including both primary and secondary vectors, as well as species from the genera Aedes and Psorophora. Differences in viral loads were observed among species. While Aedes aegypti was not found to be positive, the detection of natural YFV infection in other Aedes species raises concerns about potential virus reurbanization. Further studies are needed to clarify the role of additional mosquito species in YFV transmission in Brazil.

## 1. Introduction

Yellow fever (YF) (formerly yellow fever virus (YFV) is a severe disease caused by the arbovirus Orthoflavivirus flavi, a member of the Flaviviridae family, and remains a significant public health concern in parts of Africa and the Americas [1]. YF may cause significant morbidity and mortality rates in the human populations, as well as impacting the neotropical primate population [2]. Despite the availability of the live attenuated 17-DD vaccine, a high case fatality rate (CFR) of 40% to 60% persists, particularly in South America [1,3]. In Brazil, after the eradication of the urban YFV cycle in 1942 [4], transmitted by *Aedes aegypti* mosquitoes, YFV is maintained by a sylvatic transmission cycle involving several species of neotropical primates (NTPs) and forest canopy-dwelling mosquitoes, mainly *Haemagogus* spp. and *Sabethes* spp., and human cases are caused by a spillover process in green areas [4]. YF surveillance is based on confirmation of epizootic events through virus detection by RT-qPCR an/or immunohistochemistry in accordance with the Ministry of Health Guidelines [5].

Seasonal climatic variations significantly influence YFV transmission by affecting mosquito population dynamics and viral amplification. During the rainy season, abundant precipitation creates numerous larval habitats, while elevated temperatures and high humidity accelerate mosquito development and viral replication, leading to surges in sylvatic vectors such as *Haemagogus* and *Sabethes* spp. These conditions enhance virus amplification among non-human primates and elevate the risk of spillover to humans [6]. Conversely, in the dry season, reduced rainfall limits breeding sites and diminishes vector densities, although desiccation-resistant eggs permit a low-level virus circulation that can rapidly rebound once rains return [7].

From mid-2016 until late 2018, Brazil faced one of the largest YF outbreaks in recent decades, mainly in the southeastern region [8,9,10,11]. São Paulo state, located in southeast Brazil, is the most densely populated state in the country, containing one of the world’s largest urban conurbations [12]. A total of 875 cases of YFV in NTPs between July 2016 and November 2019 and 624 cases of YFV in humans between January 2017 and 18 November 2019 were reported. This outbreak was caused by the 1E lineage belonging to South American I (SA-I) genotype that originated in the Amazon basin [13], which was later disseminated from northern São Paulo into geographically neighboring areas of western MG and into the south of the state [14]. Some epizootic events in *Callithrix* monkeys were confirmed in large urbanized cities in proximity to urban green areas where *Haemagogus* and *Sabethes* mosquitoes were not found [15], indicating that synanthropic mosquitoes were likely involved in viral transmission in these areas. Considering that entomological investigation is a complementary tool to better understand eco-epidemiological aspects of YF after notification of suspected epizootic events, here, we describe different Aedini and Sabethini mosquitoes found to be positive for YFV by RT-qPCR and their ecological factors, showing the continuous threat of the reurbanization of YFV in Brazil.

## 2. Materials and Methods

### 2.1. Study Area

This study was conducted in the state of São Paulo, Brazil, which comprises 645 municipalities organized into 15 administrative regions. The state spans approximately 248,196,960 square kilometers and has a population of 44,749,699 inhabitants, primarily concentrated in the coastal region. São Paulo encompasses two distinct biomes, the Cerrado and the Atlantic Forest, both of which have suffered significant deforestation in recent years.

### 2.2. Epizootic Events and Mosquito Collection

Between November 2016 and June 2019, a total of 3731 mosquito pools from the Aedini tribe and Sabethes genus were formed in 192 municipalities with ongoing epizootic events and adjacent cities. Briefly, dead NTPs were detected and notified by local authorities using the Sistema de Informações de Agravos de Notificação (SINAN), and frozen carcasses were sent to Adolfo Lutz Institute for YFV detection, according to the Brazilian Ministry of Health Guidelines, as previously described [8]. Mosquitoes were then captured at ground level between 9 a.m. and 3 p.m. using entomologic nets and bottle-type manual vacuums in forested and green areas, and Nasci Aspirator in urban dwellings. After sampling, mosquitoes were frozen, transferred to cryogenic tubes, and stored in liquid nitrogen containers for transport. Identification was performed based on morphological characteristics by the Pasteur Institute (formerly the Superintendence for Control of Endemic Diseases—SUCEN). The mosquitoes were subsequently sorted into pools containing 1 to 50 individuals per pool, according to species, collection date, and location. Molecular detection for YFV was carried out on non-engorged mosquitoes (*n* = 3376) at the local reference laboratory for arthropod-borne viruses at Instituto Adolfo Lutz (IAL) in São Paulo. Pools were triturated in FastPrep-24 5G Instrument (MP Biomedicals, Irvine, CA, USA) and in Magna Lyser (Roche, Basel, Switzerland) in 1 mL of phosphate-buffered saline solution with 0.75% bovine albumin, penicillin (100 units/mL), and streptomycin (100 µg/mL). The resultant suspension was centrifuged at 1800× *g* for 15 min, and the supernatant was withdrawn and frozen at −70 °C until further use.

### 2.3. YFV RNA Detection and Statistical Analysis

Viral RNA was extracted using QIAamp Viral RNA Mini Kit following the manufacturer’s instructions (QIAGEN, Hilden, Germany). Detection of YFV RNA was performed using an RT-qPCR protocol [11]. Results with Cycle threshold (Ct) values ≥ 35 were retested. If the new result had a Ct value ≤ 38, the pool was considered positive for YFV. The Kruskal–Wallis test was conducted exclusively among YFV-positive mosquito pools to evaluate differences in viral load by mosquito species, as indicated by their Ct values. To assess differences in yellow fever virus (YFV) viral loads among different mosquito species, a Generalized Linear Model (GLM) was performed with Ct value as the dependent variable. Ct values were used as a proxy for viral load, with lower Ct values indicating higher viral loads.

The primary YFV vector, *Hg. leucocelaenus*, was set as the reference category to compare viral loads across species. The model was specified as Ct ~ Species, where Ct value was assumed to follow a Gaussian (normal) distribution with an identity link function. The analysis reports estimated mean differences in Ct values (β coefficients) for each species compared to *Hg. leucocelaenus*. To evaluate whether seasonal variation (rainy vs. dry) influenced Ct values, season was included as an additional predictor in the GLM. An interaction term (Species × Season) was also tested to assess potential species-specific seasonal effects. All *p*-values < 0.05 were considered significant. All analyses were performed using Rstudio v.2023.12.1, ggplot2 package [16].

## 3. Results

**Figure 1:**
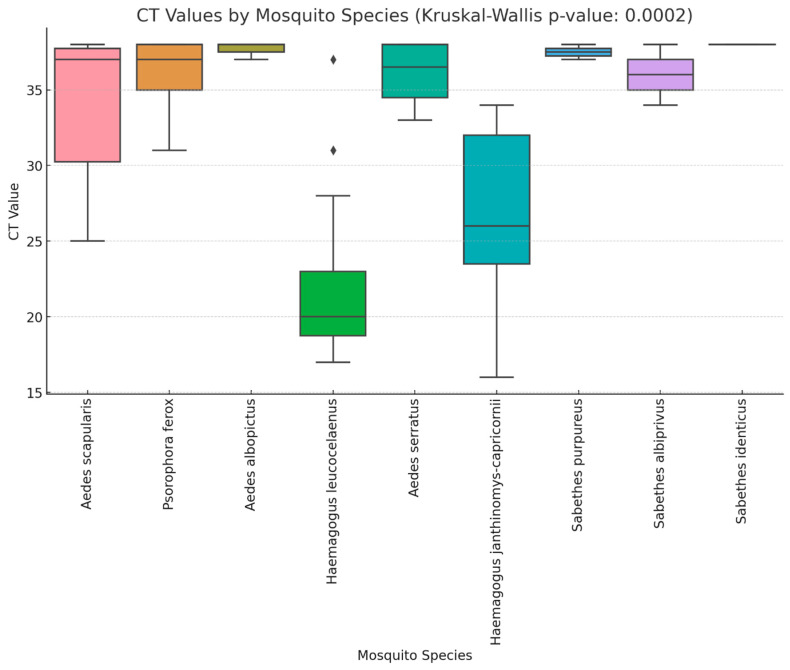
Boxplot of YFV Ct values for different Culicidae species. Analysis of Ct values among YFV-positive mosquito pools revealed significant differences in viral loads between Culicidae species, as determined by the Kruskal–Wallis test (*p* = 0.0002).

A total of 3731 mosquito pools were formed during the outbreak (Table 1), of which 46 pools (1.4%) from nine mosquitoes species tested positive for yellow fever virus (YFV), representing 22 municipalities (8.7%) (Table 2). Additionally, epizootic events were confirmed by RT-qPCR in 82 cities (Supplementary Material Figrue S1). The Ct values of the YFV-positive pools ranged from 16 to 38, with a median of 32 (Figure 1).

Among the species collected, *Aedes scapularis* accounted for 26.46% of all the mosquitoes, with 0.67% of the pools testing positive, followed by *Aedes albopictus* (21.66%, 0.41% positive) and *Psorophora ferox* (11.20%, 1.32% positive). *Haemagogus leucocelaenus* represented 8.09% of the total, with 5.83% of its pools testing positive, while *Haemagogus janthinomys/capricornii* comprised 3.4%, with 5.51% positive. Other species testing positive for YFV included *Aedes serratus* (5.72%, 2.07% positive), *Sabethes albiprivus* (2.67%, 15.78% positive), *Sabethes purpureus* (0.80%, 2.08% positive), and *Sabethes identicus* (0.74%, 1.75% positive).

The analysis of the Ct values among the YFV-positive mosquito pools revealed significant differences in the viral loads (Figure 1). The *Haemagogus* species consistently exhibited the lowest Ct values, indicating higher viral loads, while *Sa. albiprivus*, *Ae. albopictus*, *Ae. serratus*, and *Ps. ferox* had higher Ct values, suggesting lower viral loads. The distribution of the Ct values varied across species, with some species displaying a wider range, indicating heterogeneity in infection levels within the same species. While the *Ae. scapularis* pools generally showed high Ct values, two pools recorded Ct values of 25 and 28, suggesting moderate viral loads.

The Generalized Linear Model (GLM) analysis identified significant differences in Ct values among the mosquito species. *Hg. leucocelaenus* exhibited the lowest Ct values and was used as the reference species. Compared to *Hg. leucocelaenus*, *Hg. janthinomys-capricornii* showed a moderate increase in Ct values (β = 4.71, *p* = 0.039).

The mosquito species from the *Aedes*, *Psorophora*, and *Sabethes* genera exhibited significantly higher Ct values, indicating lower viral loads. *Ae. scapularis* had a β coefficient of 11.83 (*p* < 0.001), while *Ps. ferox* and *Ae. albopictus* showed β values of 13.80 and 15.67, respectively (*p* < 0.001). Among the *Sabethes* species, *Sa. purpureus*, *Sa. albiprivus*, and *Sa. identicus* exhibited the highest Ct values (β = 14.00 to 16.00, *p* < 0.01).

These results indicate species-specific differences in YFV viral loads, with the *Haemagogus* species displaying lower Ct values compared to other genera. A full summary of the GLM estimates is presented in Table 3. The effect of season (rainy vs. dry) on the Ct values was not statistically significant (*p* = 0.173). The interaction between mosquito species and season also did not significantly influence the Ct values (*p* > 0.3 for all species).

Out of the 46 positive mosquito pools, 24 (52.2%) were collected during the rainy season (18 October–4 April), and 22 (47.8%) during the dry season (Supplementary Material Figrue S2). The *Aedes* species were predominantly collected during the rainy season, whereas *Haemagogus* spp. and *Psorophora ferox* were mostly collected during the dry season. Notably, *Sa. albiprivus* and *Sa. identicus* tested positive exclusively in the dry season. All the YFV-positive mosquito pools were collected within the Atlantic Forest biome (Supplementary Material Figrue S3).

## 4. Discussion

Brazil is an endemic country for YFV, with the Amazon region acting as a source of viral diversity and dispersal across the country. Although YFV circulation has been documented in southeastern Brazil since the early 21st century, the 2016–2018 outbreak caused by the SA-I genotype, particularly in São Paulo state, was unexpected due to the high number of positive cases reported in both humans and animals [14,17]. Notably, during this outbreak, nine different species of Culicidae, including mosquitoes from the *Aedes, Psorophora*, and *Sabethes* genera, were found to be positive for YFV by RT-qPCR. All these mosquitoes were collected in the Atlantic Forest biome, where *Haemagogus leucocelaenus* act as the primary vector [18,19,20]. While *Sabethes* spp. are traditionally considered secondary vectors [3], limited information is available regarding their role in YFV transmission in this region.

Our findings confirm that the YFV viral loads varied across the Culicidae species, with *Hg. janthinomys/capricornii* and *Hg. leucocelaenus* having the highest viral loads. Notably, two pools of *Ae. scapularis* also had viral loads comparable to those of *Hg. janthinomys/capricornii*., indicating that this species may play a more relevant role in YFV transmission than previously thought. These pools were collected in Urupês on 15 February 2017, and in Araçatuba on 25 November 2016, at the municipal Zoo, yet neither location reported epizootic events at the time. The presence of YFV in these areas could be attributable to the different susceptibility of NTPs, as some *Callithryx* sp. may be less susceptible to the disease [2]. *Ae. scapularis*, which was the most abundant species collected in this study, is considered a generalist in its use of habitats, occurring in both sylvatic and human-dominated areas. Adult females are opportunistic in their behavior, feeding especially on mammals [21,22]. Considering the wide host breadth and feeding habitats, coupled with synanthropic adaptions, it is possible that *Ae. scapularis* may be an important bridge vector for human and animal viruses. Thus, our data suggest that this species may have played a secondary role in the YF outbreak.

*Sabethes* mosquitoes were observed to have low abundance, distribution, and infection rates, suggesting a local or secondary role during the 2016–2018 outbreak in the Brazilian southeastern region [18]. In our study, this genus accounted for 11.5% (*n* = 430 pools) of the collected mosquitoes and for 10.9% (*n* = 5/46) of the positive pools, all exhibiting high Ct values, indicative of low viral loads. Similarly, during the 2009 YF outbreak in São Paulo, YFV was only isolated from a single pool of *Hg. leucocelaenus* in Buri, despite the collection of *Sa. chloropterus*, *Sa. purpureus*, and *Sa. undosus* in the same area [19]. However, the absence of an RT-qPCR analysis in that study may explain the lack of positive detections among Sabethini mosquitoes. Conversely, during a YF epidemic and epizootic in Misiones, a northeastern province of Argentina, YFV was successfully isolated in a cell culture from pools of *Sabethes albiprivus* [23]. This viral isolation indicates high viral loads, contrasting with the low viral loads observed in the *Sabethes* specimens from the Atlantic Forest.

*Sa. chloropterus* has been identified as the primary YF vector during the dry season in the Cerrado biome of Minas Gerais [24]. In Espírito Santo, where the sylvatic YF cycle was first described in Brazil, *Sa. chloropterus*, *Sa. soperi*, *Sa. identicus*, *Aedes aureolineatus*, and *Shannoniana fluviatilis* were noted for their secondary roles in YFV transmission [25]. Additionally, *Sa. albiprivus* from Rio de Janeiro demonstrated high vector competence when inoculated with Brazilian YFV strains [26]. To better elucidate the role of *Sabethes* mosquitoes in the YF transmission cycle within São Paulo state, where the virus has now been established [27], additional studies are required.

Regarding the *Aedes* genus, earlier studies suggested that Brazilian *Ae. aegypti* mosquitoes might not favor the establishment of an urban cycle of YF [28]. However, a more recent study demonstrated that both anthropophilic mosquitoes, *Ae. aegypti* and *Ae. albopictus*, are highly susceptible to American and African YFV strains [26]. In 2018, in Minas Gerais state, a single *Ae. albopictus* mosquito pool tested positive for YFV [29]. In our surveillance study, *Ae. albopictus* was the second most frequent species collected, accounting for 21.66% of the total, with three pools testing positive for YFV, all of which exhibited low viral loads. No *Ae. aegypti* mosquitoes were found to be positive. Despite the high number of human infections during the outbreak, no urban YF cases were reported. Given that YFV has demonstrated potential for adaptation to *Ae. albopictus* and can be transmitted between NTPs [30,31] our findings underscore a potential threat to the endemic areas in South America where these mosquitoes are present. With its widespread distribution and ecological plasticity, *Ae. albopictus* could serve as a bridge vector, facilitating virus transmission between urban environments and rural areas.

One objective of this study was to assess whether seasonal variation (rainy vs. dry) influenced YFV viral loads in mosquitoes. Despite previous reports showing seasonal peaks in mosquito abundance and transmission during rainy periods [32] our results indicate that season was not a significant predictor of Ct values, suggesting that once a mosquito is infected, viral replication remains stable. Sacchetto and collaborators reported viral persistence during the non-epidemic dry season in NTPs collected in Belo Horizonte, Minas Gerais state [33]. These results show the importance of continuous surveillance, regardless of seasonal variations.

Our study has some limitations. Specifically, our study involved triturating whole mosquitoes instead of processing solely the salivary gland. Additionally, the contents of the mosquitoes’ digestive systems—whether engorged or not—were assessed solely through visual examination, and some of the positive results could have come from residual blood feeding. Nevertheless, the data obtained in the present study are relevant, as monitoring of virus circulation and characterizing vectors are fundamental elements for understanding the dynamics of vector-borne viruses, providing new insights for the establishment of control strategies and to prevent the risk of the re-urbanization of YFV. More studies of vectorial competence, mainly in *Ae. scapularis*, are needed, as our results suggest a possible role of *Ae. scapularis* in the YFV cycle in the Atlantic Forest.

## Figures and Tables

**Table 1:** Number of Culicidae pools tested for YFV and positive samples with percentages.

Species	N	%	Positive	%Pos
*Aedes scapularis*	893	26.46	6	0.67
*Aedes albopictus*	731	21.66	3	0.41
*Psorophora ferox*	378	11.20	5	1.32
*Haemagogus leucocelaenus*	274	8.09	16	5.83
*Aedes serratus*	193	5.72	4	2.07
*Aedes aegypti*	148	4.39	0	0
*Haemagogus janthinomys/capricornii*	127	3.4	7	5.51
*Sabethes purpureus*	96	2.84	2	2.08
*Sabethes glaucodaemon*	94	2.79	0	0
*Aedes terrens*	72	2.13	0	0
*Sabethes identicus*	57	1.69	1	1.75
*Sabethes albiprivus*	47	1.39	2	4.26
*Sabethes chloropterus*	46	1.23	0	0
*Psorophora albigenu*	31	0.83	0	0
*Sabethes intermedius*	28	0.83	0	0
*Psorophora albipes*	27	0.80	0	0
*Psorophora (Jan.)* sp.	17	0.50	0	0
*Sabethes belisarioi*	16	0.47	0	0
*Aedes argyrothorax*	13	0.39	0	0
*Psorophora* sp.	11	0.33	0	0
*Sabethes* sp.	11	0.30	0	0
*Aedes* sp.	9	0.27	0	0
*Sabethes undosus*	9	0.27	0	0
*Sabethes tridentatus*	8	0.24	0	0
*Psorophora lutzii*	6	0.18	0	0
*Sabethes soperi*	6	0.18	0	0
*Sabethes whitmani*	6	0.18	0	0
*Howardina fulvithorax*	6	0.18	0	0
*Culex* sp.	4	0.12	0	0
*Sabethes undosus aff.*	3	0.09	0	0
*Aedes fluviatilis*	2	0.06	0	0
*Culex quinquefaciatus*	2	0.06	0	0
*Limatus* sp.	1	0.03	0	0
*Psorophora lanei*	1	0.03	0	0
*Sabethes belisarioi aff.*	1	0.03	0	0
*Sabethes petrochiae*	1	0.03	0	0
*Sabethes shannoni*	1	0.03	0	0

**Table 2:** YFV-positive mosquito species and number of mosquitoes per pool collected in São Paulo State, 2016–2018.

Pool Number	Species (n° per pool)	Local	Ct Value	Date	Season
732	*Aedes albopictus* (2)	Jundiaí	38	28 August 2018	Dry
3777	*Aedes albopictus* (2)	Itariri	38	16 January 2019	Rainy
4231	*Aedes albopictus* (6)	Pereira Barreto	37	16 January 2019	Rainy
443	*Aedes scapularis* (8)	Urupes	25	26 November 2016	Rainy
1415	*Aedes scapularis* (1)	Araçatuba	28	25 November 2016	Rainy
2348	*Aedes scapularis (3)*	Sao Paulo	37	19 February 2018	Rainy
4232	*Aedes scapularis* (1)	Pereira Barreto	37	16 January 2019	Rainy
4234	*Aedes scapularis* (1)	Pereira Barreto	38	16 January 2019	Rainy
4238	*Aedes scapularis* (3)	Sao Paulo	38	16 January 2019	Rainy
2198	*Aedes serratus* (1)	Jarinu	33	12 February 2019	Rainy
4233	*Aedes serratus* (1)	Pereira Barreto	38	16 January 2019	Rainy
4276	*Aedes serratus* (1)	Monteiro Lobato	35	16 January 2019	Rainy
4449	*Aedes serratus* (1)	Iguape	38	25 March 2019	Rainy
2438	*Haemagogus janthinomys-capricornii* (1)	Mairipora	33	23 January 2018	Rainy
2572	*Haemagogus janthinomys-capricornii* (1)	Valinhos	31	4 September 2018	Dry
2577	*Haemagogus janthinomys-capricornii* (12)	Valinhos	34	17 September 2018	Dry
3551	*Haemagogus janthinomys-capricornii* (7)	Igarata	16	23 May 2018	Dry
3689	*Haemagogus janthinomys-capricornii* (1)	Sao José dos Campos	25	4 July 2018	Dry
3766	*Haemagogus janthinomys-capricornii* (4)	Caçapava	22	25 June 2018	Dry
4298	*Haemagogus janthinomys-capricornii* (2)	Monteiro Lobato	26	25 March 2019	Rainy
2152	*Haemagogus leucocelaenus* (3)	Caieras	23	16 April 2019	Dry
2163	*Haemagogus leucocelaenus* (11)	Guarulhos	21	14 December 2018	Rainy
2322	*Haemagogus leucocelaenus* (1)	Jarinu	37	3 May 2018	Dry
2377	*Haemagogus leucocelaenus* (2)	Jarinu	20	30 January 2018	Rainy
3268	*Haemagogus leucocelaenus* (15)	Sao Paulo	19	20 December 2017	Rainy
3270	*Haemagogus leucocelaenus* (24)	Sao Paulo	18	20 December 2017	Rainy
3318	*Haemagogus leucocelaenus* (8)	Sao José dos Campos	28	10 October 2018	Dry
3514	*Haemagogus leucocelaenus (11)*	Piedade	31	10 October 2018	Dry
3521	*Haemagogus leucocelaenus* (7)	Jacarei	20	4 September 2018	Dry
3530	*Haemagogus leucocelaenus* (7)	Jacarei	18	28 May 2018	Dry
3541	*Haemagogus leucocelaenus* (5)	Igarata	19	28 May 2018	Dry
3552	*Haemagogus leucocelaenus (9)*	Igarata	20	23 May 2018	Dry
3687	*Haemagogus leucocelaenus* (6)	Sao José dos Campos	23	4 July 2018	Dry
4272	*Haemagogus leucocelaenus* (7)	Monteiro Lobato	17	16 January 2019	Rainy
4275	*Haemagogus leucocelaenus* (15)	Monteiro Lobato	17	16 January 2019	Rainy
4297	*Haemagogus leucocelaenus* (2)	Monteiro Lobato	21	25 March 2019	Rainy
465	*Psorophora ferox* (14)	Pontalinda	38	21 August 2018	Dry
4188	*Psorophora ferox* (2)	Jacupiranga	31	10 December 2018	Rainy
4273	*Psorophora ferox* (1*)*	Monteiro Lobato	37	16 January 2019	Rainy
4568	*Psorophora ferox* (1*)*	Sarapui	38	21 May 2018	Dry
5077	*Psorophora ferox* (2)	Iporanga	35	25 April 2019	Dry
3542	*Sabethes albiprivus* (1)	Igarata	38	28 May 2018	Dry
3769	*Sabethes albiprivus* (3)	Caçapava	34	25 June 2018	Dry
3543	*Sabethes identicus* (6)	Igarata	38	28 May 2018	Dry
3491	*Sabethes purpureus* (2)	Sao Miguel Arcanjo	37	4 September 2018	Dry
4279	*Sabethes purpureus* (1)	Monteiro Lobato	38	16 January 2019	Rainy

**Table 3:** GLM statistics for YFV-positive mosquitoes by species.

Species	Estimate (β)	SE	95% CI	*p*-Value
Intercept (*Haemagogus leucocelaenus*)	22	1.26	(19.53, 24.47)	<0.001
*Aedes scapularis*	11.83	2.41	(7.11, 16.56)	<0.001
*Psorophora ferox*	13.8	2.58	(8.74, 18.86)	<0.001
*Aedes albopictus*	15.67	3.17	(9.46, 21.88)	<0.001
*Aedes serratus*	14	2.81	(8.48, 19.52)	<0.001
*Haemagogus janthinomys/capricornii*	4.71	2.28	(0.24, 9.19)	0.039
*Sabethes purpureus*	15.5	3.78	(8.10, 22.90)	<0.001
*Sabethes albiprivus*	14	3.78	(6.60, 21.40)	<0.001
*Sabethes identicus*	16	5.19	(5.83, 26.17)	0.002

## Data Availability

The data presented in this study are available on request from the corresponding author.

## Supplementary Materials

The following supporting information can be downloaded at https://www.mdpi.com/article/10.3390/tropicalmed10050118/s1: Figure S1: Yellow fever in São Paulo State. Municipalities with dots indicate YFV epizootic events. Mosquito-positive pools are represented by their respective counts by municipality. Maps were created using QGIS v.2.14.9 Essen.; Figure S2: Positive mosquito pools collected in São Paulo state by season.; Figure S3: Positive mosquito species collected in São Paulo state by biome (Atlantic Forest and Cerrado). Maps were created using QGIS v.2.14.9 Essen.
