# Supplementary material for: Yellow Fever Virus (YFV) Detection in Different Species of Culicids Collected During an Outbreak in Southeastern Brazil, 2016–2019
Source: Trop Med Infect Dis. 2025 Apr 24;10(5):118. doi: 10.3390/tropicalmed10050118 (PMC12115348; doi:10.3390/tropicalmed10050118)

Yellow Fever virus (YFV) detection in different species of culicids collected during an outbreak in Southeastern Brazil, 2016-2019

Supplementary material

Figure S1: Yellow fever in São Paulo State. Municipalities with dots indicate YFV epizootic events. Mosquito-positive pools are represented by their respective counts by municipality. Maps were created using QGIS v.2.14.9 Essen.

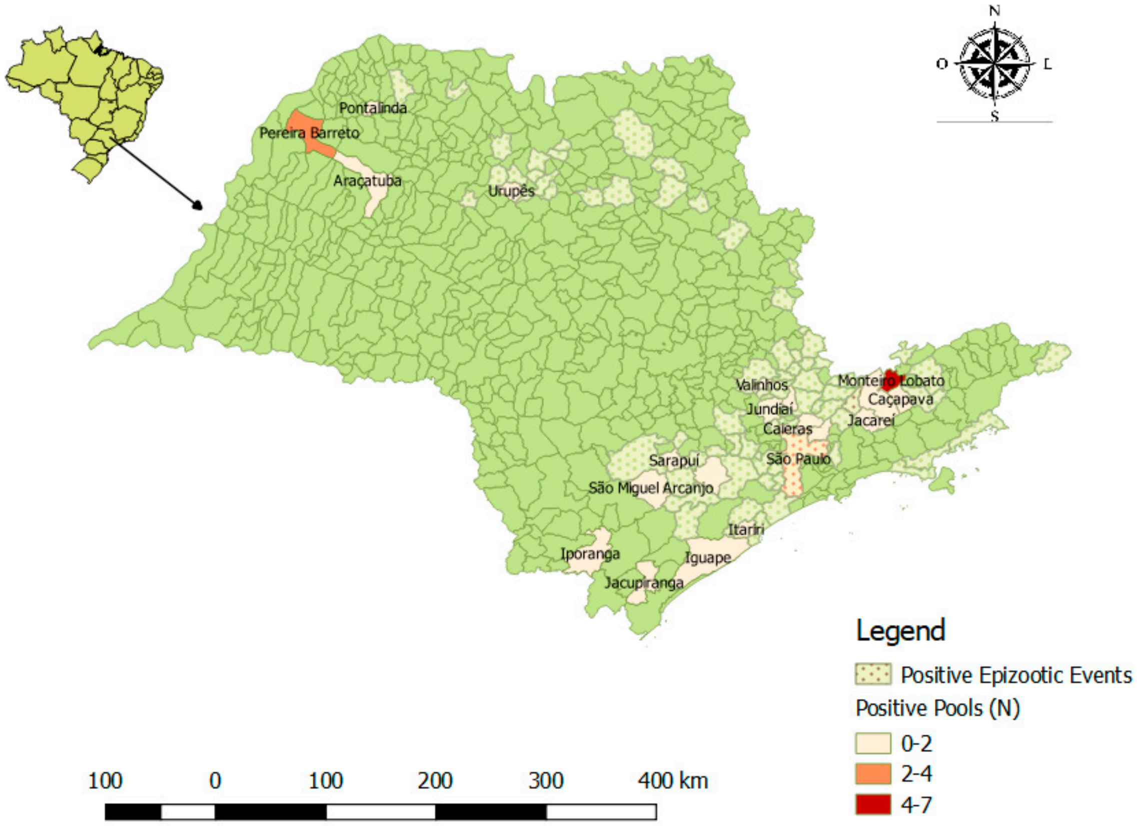

Figure S2: Positive mosquito pools collected in São Paulo state by season.

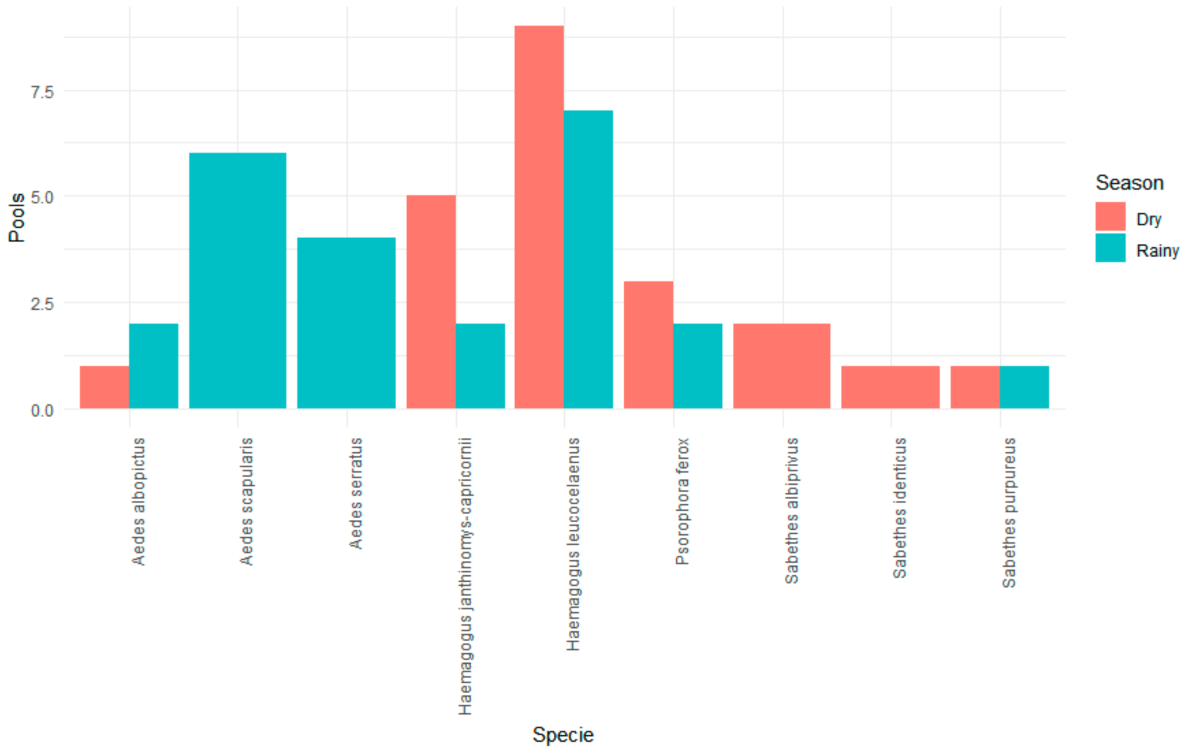

Figure S3 Positive mosquito species collected in São Paulo state by biome (Atlantic Forest and Cerrado). Maps were created using QGIS v.2.14.9 Essen.

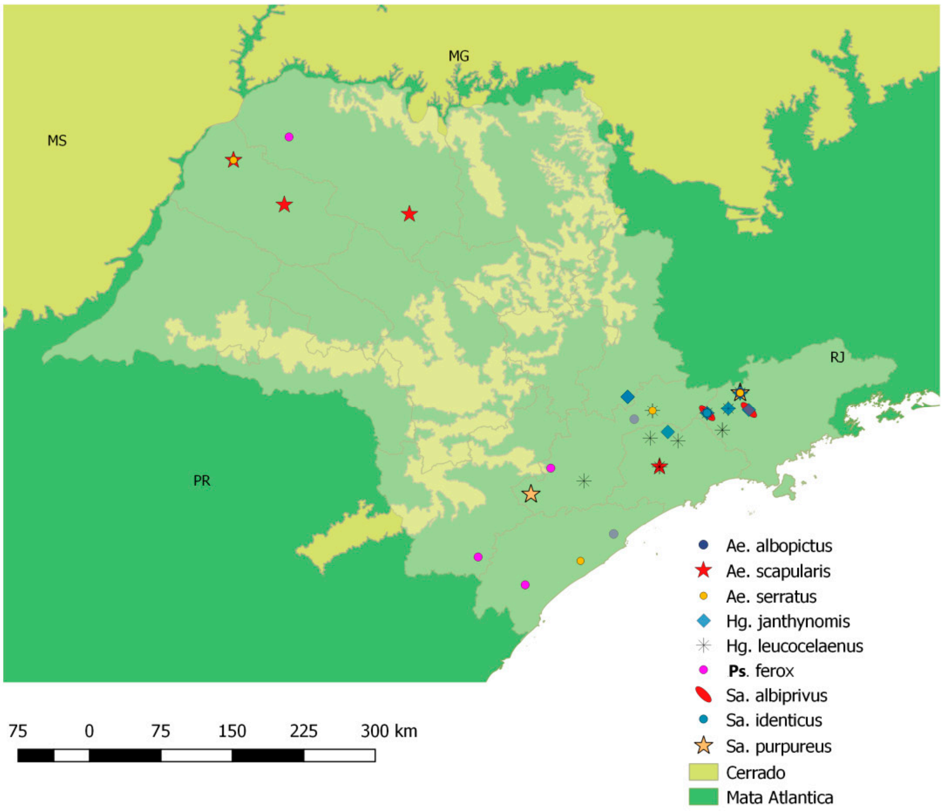

Supplement: Supplementary file 1 [file tropicalmed-10-00118-s001.zip › tropicalmed-3567129-supplementary.pdf]
